# Novel Carbon Nanoparticles Derived from Biodiesel Soot as Lubricant Additives

**DOI:** 10.3390/nano9081115

**Published:** 2019-08-03

**Authors:** Chuan Li, Mingling Li, Xinyun Wang, Weimin Feng, Qiangqiang Zhang, Bo Wu, Xianguo Hu

**Affiliations:** 1 School of Chemistry and Material Engineering, Chaohu University, Hefei 238000, China; 2 School of Mechanical Engineering, Hefei University of Technology, Hefei 230009, China

**Keywords:** biodiesel soot, carbon nanoparticles, onion-like, lubricant additive

## Abstract

The objective of this study was to investigate the roles and tribological mechanisms of onion-like carbon nanoparticles derived from biodiesel soot (BDS) when applied in water (H_2_O) and liquid paraffin (LP). In this study, we prepared nitric acid-treated BDS (NA-BDS) as an additive to H_2_O and NA-BDS modified with oleylamine (NA-BDS-OLA) as an additive to LP. Raman spectroscopy, field-emission transmission electron microscopy, Fourier transform infrared spectroscopy, and zeta potentiometry were used to characterize the results of the nitric acid treatment and oleylamine modification. The tribological behaviors and corresponding mechanisms of the new onion-like carbon nanoparticles were evaluated using a ball-on-disc reciprocating tribometer, as well as field-emission scanning electron microscopy, three-dimensional laser scanning microscopy, and Raman spectroscopy. The results indicated that the additives NA-BDS and NA-BDS-OLA, which were onion-like carbon nanoparticles with sizes ranging from 35 to 40 nm, enhanced the antiwear and friction reduction properties of H_2_O and LP, respectively. Through tribo-mechanisms, these types of soot can serve as spacers and ball bearings between the rubbing surfaces. Moreover, exfoliation under a high load as a result of the formation of a graphitic layer facilitates easy shearing.

## 1. Introduction

With changes in the global climate and with the growing energy demand, there has been an increased interest in renewable fuels for the replacement of petroleum. Biodiesel is a potential renewable fuel source. Biodiesel (fatty acid alkyl ester) can be obtained via the transesterification process [1]. It exhibits characteristics comparable to those of petroleum diesel, as well as a low sulfur amount, low aromatics, low viscosity, a high flash point, high lubricity, and a high cetane number [2]. Although biodiesel has a higher oxygen amount than petroleum diesel, which can enhance its combustion efficiency, biodiesel soot (BDS) is still emitted [3,4].

Soot can contaminate the lubricating oil within the sump as a result of the blow-by gasses [5]. Soot in the lubricating oil can increase the viscosity of the oil and affect its tribological properties. Salehi et al. [6] found that rapid tribofilm formation through the addition and removal of ZDDP (Zinc Dialkyl Dithiophosphates)by soot particles results in severe wear, which can be attributed to the corrosive–abrasive mechanism. Green et al. [7] concluded that a larger soot amount in the lubricating oil generates more wear, which is mainly due to the abrasive process. However, soot also has potential as a lubricant additive. Guo et al. [8] reported that the anti-wear and friction-reduction properties of PAO (Polyalphaolefin) 4 oil were improved by adding 0.01 wt % diesel soot. Additionally, carbon nano-onions have been widely studied as lubrication additives because they can improve the tribological properties of oils. Joly-Pottuz et al. [9] found that carbon nano-onions have no dangling bonds on their surface and can therefore easily slide and even roll on a friction surface. Hirata et al. [10] reported that the closed structure of carbon nano-onions provides good mechanical strength. BDS contains short grapheme segments of high tortuosity [11,12], which exhibit great potential for the development of onion-like carbon nanoparticles. However, to the best of our knowledge, few reports have described onion-like carbon nanoparticles derived from BDS as a lubricant additive.

At present, real engine soot is expensive and not widely available because it can only be produced by running an engine for a long time under suboptimal conditions [13]. Thus, engine soot alternatives (such as carbon black and self-prepared soot) are typically used to research the effect of engine soot on the lubricating oils [14,15]. Clague et al. reported that diesel engine soot and carbon black exhibited similar primary particle sizes and internal structures of particulates [16]. Ferraro et al. used various techniques to compare carbon black and diesel engine soot and found that carbon black was an appropriate substitute for engine diesel soot [17]. Alternatively, self-prepared soot can be used as an engine-soot alternative. Self-prepared soot was prepared via the combustion of fuel at a normal temperature and the atmospheric pressure [18]. Hu et al. compared the differences between carbon black and self-prepared biomass-oil soot. Differences were observed in the elemental composition and the functional groups. However, self-prepared biomass-oil soot is similar in size, morphology, and internal structure to carbon black [19]. Studies have indicated that self-prepared BDS has discontinuous graphitic layers and that the graphitic layers are surrounded by an amorphous phase [20]. This structure is similar to that of real BDS from a diesel engine [21,22]. Thus, self-prepared BDS can be used as an alternative to real BDS. 

In this study, onion-like carbon nanoparticles derived from BDS were prepared. The tribological properties of nitric acid-treated BDS (NA-BDS) as an additive in water (H_2_O) and NA-BDS modified with oleylamine (NA-BDS-OLA) as an additive in liquid paraffin (LP) were investigated. The corresponding tribological mechanisms were also examined. Thus, the objective of this study was to provide a new path for recycling carbon resources from engine emissions and for reducing environmental pollution from fuel combustion.

## 2. Materials and Methods 

### 2.1. Sample Preparation

All reagents were analytical-grade. Methanol (Sinopharm Chemical Reagent Co., Ltd., Shanghai, China), Oleylamine (Aladdin Industrial Co., Ltd., Shanghai, China), Ethanol (China Sun Specialty Products Co., Ltd., Changshu, China), Nitric acid (68%, Sinopharm Chemical Reagent Co., Ltd., Shanghai, China). And soybean oil was obtained from China Oil & Food Stuffs Corporation (Beijing, China).Ultrapure water obtained from a water purification system (UPT-11-10T, Sichuan Ulupure Super Pure Technology Co., Ltd., Chengdu, China) was used in all experiments. Biodiesel was synthesized through transesterification between methanol and soybean oil using a calcium-based catalyst. The physical properties are presented in Table 1. The procedure for collecting BDS was as follows [23,24]: (1) First, 80 cm^3^ of biodiesel was poured into a 100 cm^3^ ceramic crucible, and then the ceramic crucible was placed on an iron stand. (2) Clean glass slides (BDS capturing slides) were used as substrates to collect the BDS. The glass slides were clamped on the upper part of the iron stand, above the ceramic crucible. When the biodiesel was ignited, the slide was kept over the flame at a distance of 2 cm. (3) As the biodiesel burned, the BDS in the flame was deposited onto the slide surface. When the biodiesel in the ceramic crucible was completely burned and a BDS layer with a thickness of a few micrometers was deposited on the glass slide, the BDS layer was carefully removed using a laboratory spoon and collected in a glass bottle. (4) Finally, the collected BDS was dried at 120 °C for 3 h and ground into a fine granular powder. Burning 1 kg of biodiesel yielded 13–18 g of BDS.

A total of 0.2 g of BDS was suspended in 30 g of concentrated nitric acid (68%, analytical reagent) and refluxed for 20 h in an oil bath maintained at 80 °C. After repeated centrifugation and washing with ultrapure water, it was then dried at 120 °C for 10 h. The product was denoted as NA-BDS. Then, 0.1 g of NA-BDS was refluxed in 5 g of oleylamine (OLA) at 110 °C for 12 h. The mixture was purified through repeated washings with ethanol using centrifugation. Finally, it was dried at 120 °C for 5 h. The product was denoted as NA-BDS-OLA. As additives, BDS and NA-BDS (at concentration levels of 0.1, 0.2, and 0.3 wt %) were added to the H_2_O. Moreover, BDS and NA-BDS-OLA (at concentration levels of 0.2, 0.3, and 0.4 wt %) were added to the LP. The mixtures were then distributed using a glass rod for 15 min, followed by magnetic stirring for 1 h to reduce any deviations in the experiment.

### 2.2. Tribological Testing and Characterization

**Figure 1:**
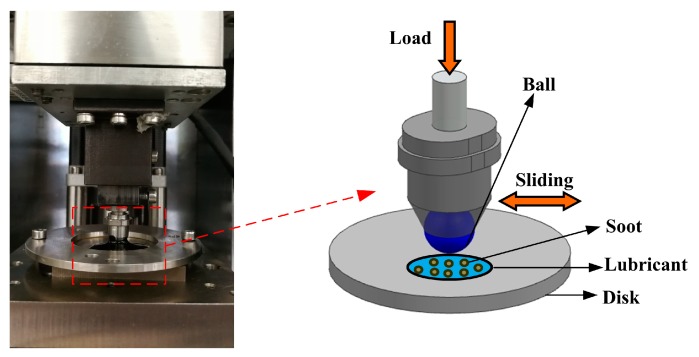
Configuration of the friction pairs of the ball-on-disk reciprocating tribometer.

Tribological testing was performed using a ball-on-disc reciprocating tribometer (CFT-I, Lanzhou Zhongke Kaihua Technology Development Co. Ltd., Lanzhou, China). The upper ball samples (Φ 6 mm, surface roughness of *R*_a1_ = 0.02 µm) and lower disk samples (50 mm × 50 mm × 5 mm, surface roughness of *R*_a2_ = 0.5 µm) were made of GCr15 steel with a hardness of 62–65 HRC (Rockwell hardness value). The lower disk samples were fixed, and the upper ball samples were slid in a reciprocating manner with a sliding distance of 5 mm. The friction pairs of the ball-on-disk reciprocating tribometer are illustrated in Figure 1. The wear rate is estimated by Archard’s equation. As the initial roughness of the lower disk samples was high, the wear volume of disc samples was very difficult to assess [6]. Therefore, the wear volume of upper ball samples was used to calculate the wear rate. In order to determine the wear rate of the upper ball samples, the following Equations (1), (2), and (3) were used [25]:

*V* = π × *h* × (0.75*d*^2^ + *h*^2^)/6, (1)

*h* = *R* − (*R*^2^ − 0.25*d*^2^)^0.5^, (2)

*K_B_* = *V*/(*L* × *F*), (3)

where *K_B_* represents ball wear rate (in mm^3^/N·m), *d* represents the diameter of the wear scar of the upper ball (in mm), *h* represents the height of the wear of the upper ball (in mm), *R* represents the radius of the upper ball (in mm), *V* represents the wear volume of the upper ball sample (in mm^3^), *L* represents the sliding distance (in m), and *F* represents the applied load (in N). Each tribological test was repeated three times to obtain a standard deviation and to reduce human error.

For the ball-on-disc contact, the Hertz contact diameter and the contact stress were calculated using Hertz’s theory [26]:

(4) $$a=2\times {\left(\frac{2}{3}\times \frac{W\times R}{E^{\prime }}\right)}^{\frac{1}{3}},$$

(5) $$P=\frac{4\times W}{\pi \times a^{2}},$$

where *a* represents the Hertz contact diameter, *W* represents the normal load (= 20 N, 50 N, 100 N), *R* represents the equivalent radius of curvature (3 mm), and *E*′ represents the effective elastic modulus (233 GPa). Thus, the Hertz diameter was 1.11 × 10^−4^ m, 1.51 × 10^−4^ m, and 1.90 × 10^−4^ m, and the contact stress was 2 GPa, 2.8 GPa, and 3.5 GPa.

The λ was calculated using Dowson and Hamrock’s minimum film thickness formula:

(6) $$h_{\min}=3.63\times R\times \frac{G^{*0.49}\times U^{*0.68}}{W^{*0.073}}\left(1-e^{-0.68k}\right),$$

(7) $$\lambda =\frac{h_{\min}}{\sqrt{Ra_{1}^{2}+Ra_{2}^{2}}},$$

(8) $$G^{*}=\alpha \times E^{\prime },$$

(9) $$U^{*}=\frac{\eta_{0}\times U}{E^{\prime }\times R},$$

(10) $$W^{*}=\frac{W}{E^{\prime }\times R^{2}},$$

where *R* represents the equivalent radius of curvature (= 3 mm), *k* is the elliptical parameter (≈ 1), *η*_0_ represents the dynamic viscosity of water or LP (1 × 10^−3^ or 100.2 × 10^−3^ Ns/m^2^, respectively), *U* represents the sliding speed (= 50 mm/s), *E*′ represents the effective elastic modulus (= 233 GPa), *α* is the coefficient of viscosity–pressure of water and liquid paraffin, respectively, (2.2 × 10^−8^ m^2^/N or 2 × 10^−8^ m^2^/N) [27,28], and *R*_a1_ and *R*_a2_ represent the surface roughness values of the ball and disc, respectively (*R*_a1_ = 0.02 µm, *R*_a2_ = 0.5 µm).

The maximum value of *λ* (*W* = 20 N) was calculated to be 0.0019 and 0.0418 under water and LP lubrication, respectively, for the test conditions. This confirms that the sliding tests were conducted in the boundary lubrication regime.

The morphologies of BDS, NA-BDS, and NA-BDS-OLA were examined using Raman spectroscopy (HR Evolution, Horiba Jobin Yvon, Paris, France), field-emission transmission electron microscopy (FETEM, JEM-2100F, JEOL, Tokyo, Japan), and Fourier transform infrared (FTIR) spectroscopy (Nicolet6700, Thermo Nicolet, Madion, WI, USA). The particle-size distributions of BDS and NA-BDS in H_2_O, as well as those of BDS and NA-BDS-OLA in LP, were characterized using a zeta potentiometer (Nano-ZS90, Malvern, Worcestershire, UK). After friction and wear tests, the friction surfaces were inspected and analyzed using field-emission scanning electron microscopy (FESEM, SU8010, Hitachi, Tokyo, Japan), three-dimensional laser scanning microscopy (VK-X100, Keyence, Osaka, Japan), and Raman spectroscopy.

## 3. Results and Discussion

### 3.1. Characterization

**Figure 2:**
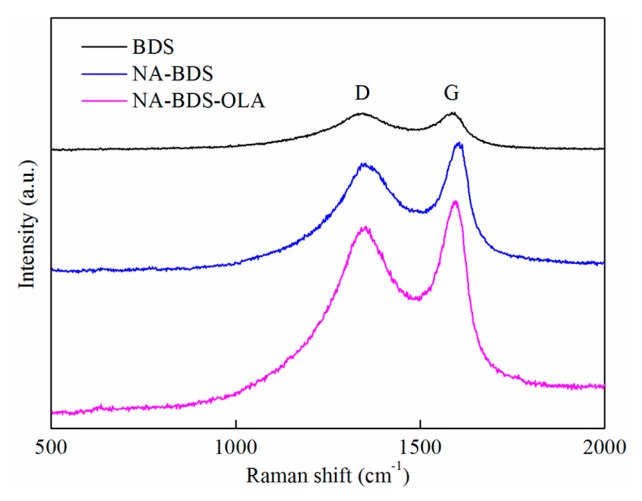
Raman spectra of biodiesel soot (BDS), nitric-acid-treated BDS (NA-BDS), and NA-BDS modified with oleylamine (NA-BDS-OLA).

To investigate the effects of nitric acid treatment and oleylamine (OLA) modification on BDS, Raman spectroscopy, FETEM, FTIR spectroscopy, and particle-size distribution analyses were conducted. The Raman spectra of BDS, NA-BDS, and NA-BDS-OLA are presented in Figure 2. The Raman spectra exhibited well-defined D and G peaks. The D peak (at ~1346 cm^−1^) is attributed to the defects and disordered graphitic lattices. The G peak (at ~1589 cm^−1^) is attributed to the graphitic lattices [29,30]. In general, the I_D_/I_G_ ratio increased with the increase in the degree of disorder. A comparison of the Raman spectra of BDS and NA-BDS indicated that the I_D_/I_G_ ratio of the NA-BDS (2.546) was lower than that of BDS (2.956). The I_D_/I_G_ ratio of NA-BDS-OLA (2.559) was similar to that of NA-BDS (2.546). The spectroscopic results indicated that nitric acid treatment improved the order of BDS.

**Figure 3:**
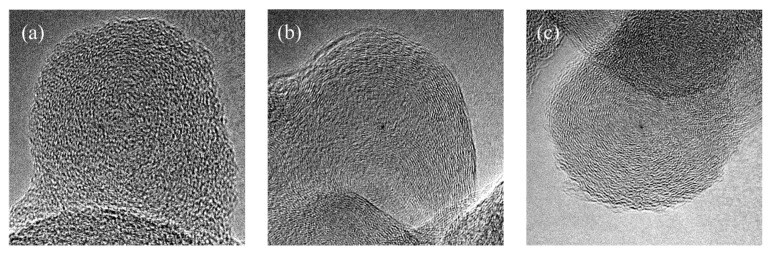
Field-emission transmission electron microscopy (FETEM) micrographs of (**a**) BDS, (**b**) NA-BDS, and (**c**) NA-BDS-OLA.

The Raman results were complemented with FETEM observations of BDS, NA-BDS, and NA-BDS-OLA, as shown in Figure 3. The diameters of BDS, NA-BDS, and NA-BDS-OLA were approximately 35–40 nm. For BDS, short, disconnected, and concentrically-oriented graphitic layers were observed, as shown in Figure 3a. However, these graphitic layers were surrounded by amorphous phases. As shown in Figure 3b,c, compared with BDS, the apparent growth of highly curved and discontinuous graphitic layers was observed in NA-BDS and NA-BDS-OLA, respectively, which were structurally similar to onion-like carbon. A statistical evaluation of the FETEM micrographs indicated that the degree of graphitization was higher for NA-BDS and NA-BDS-OLA than that for BDS, in agreement with the Raman results [31].

**Figure 4:**
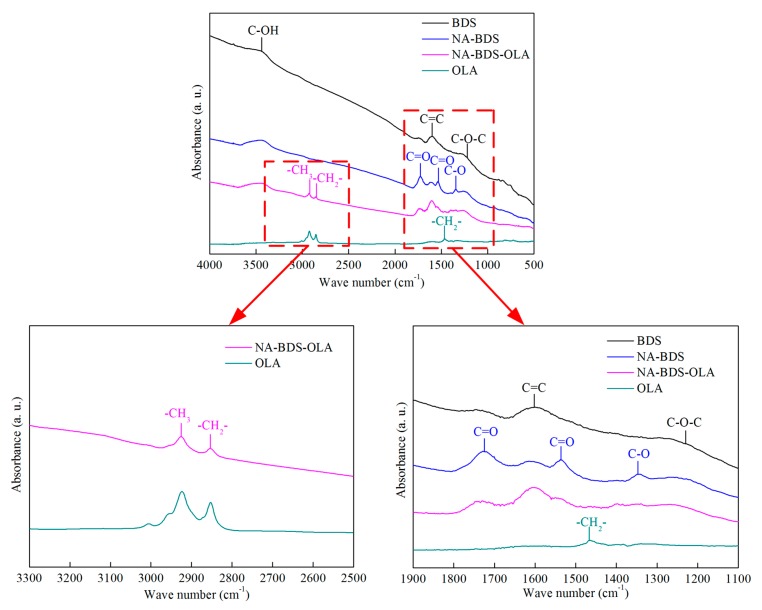
Fourier transform infrared (FTIR) spectra of oleylamine (OLA), BDS, NA-BDS, and NA-BDS-OLA.

As shown in Figure 4, the functional groups on the surfaces of OLA, BDS, NA-BDS, and NA-BDS-OLA were investigated using FTIR spectroscopy. For BDS, the three main peaks at 1221, 1602, and 3443 cm^−1^ are ascribed to the C–O–C, C=C, and C–OH stretching vibrations, respectively. For NA-BDS, the peaks at (1535 cm^−1^ and 1726 cm^−1^) and 1347 cm^−1^ are attributed to the stretching vibrations of C=O and C–O, respectively, indicating that strong (–COOH) polar groups were introduced into the NA-BDS through the nitric acid treatment [31]. For OLA, the main peaks in the spectrum are related to –CH_2_– (1465 cm^−1^ and 2853 cm^−1^) and –CH_3_ (2923 cm^−1^). Moreover, for NA-BDS-OLA, the peaks at 2853 and 2923 cm^−^^1^ are associated with –CH_2_– and –CH_3_ stretching vibrations, respectively, indicating that the surface of NA-BDS-OLA contained long-carbon-chain lipophilic groups owing to the OLA treatment [32]. This was because of the many active hydroxyl and carboxyl groups of NA-BDS which can adsorb and react with OLA. Interestingly, the peak shape of C–H under NA-BDS-OLA became narrower than OLA, indicating that the C–H content of NA-BDS-OLA was smaller than OLA.

**Figure 5:**
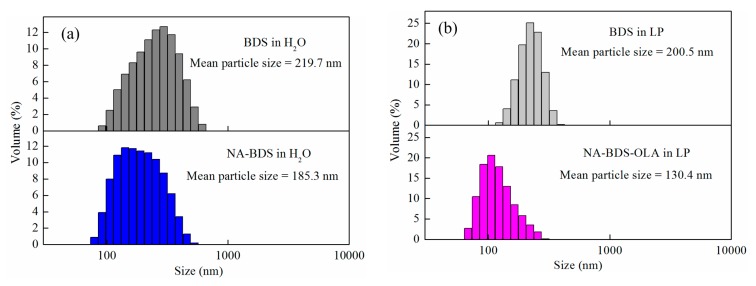
Particle-size distributions of (**a**) BDS and NA-BDS in H_2_O, and (**b**) BDS and NA-BDS-OLA in liquid paraffin (LP).

The particle-size distributions of the different soot samples are presented in Figure 5. As shown in Figure 5a, the particle size of BDS in H_2_O ranged from 91.3 to 615.1 nm, with a mean value of 219.7 nm. Compared with BDS, the mean particle size of NA-BDS in H_2_O was reduced to 185.3 nm, whereas the particle size of NA-BDS in H_2_O ranged from 78.8 to 531.2 nm. This phenomenon was due to the oxidation of BDS, which increased the amount of hydrophilic functional groups in NA-BDS [31], and stabilized the material in H_2_O. As indicated by Figure 5b, the mean particle size decreased from 200.5 nm (between 122.4 and 396.1 nm for BDS in LP) to 130.4 nm (between 68.1 and 295.3 nm for NA-BDS-OLA in LP), which is ascribed to the introduction of functional groups with high lipophilicity on the surface of NA-BDS-OLA [33]. These results agree well with the FTIR measurements.

### 3.2. Tribological Performance of Soot Samples in H_2_O

**Figure 6:**
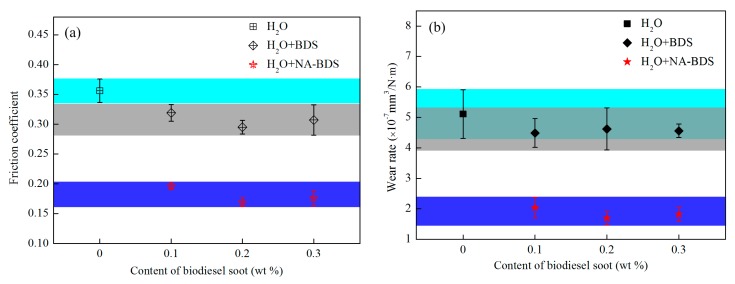
Evolutions of the (**a**) average friction coefficient and (**b**) wear rates for lubrication with different amounts of BDS and NA-BDS in H_2_O (load = 50 N, speed = 50 mm/s, duration = 30 min).

The evolutions of the average friction coefficient and wear rates for lubrication with different amounts of two types of soot in H_2_O are plotted in Figure 6. Clearly, both BDS and NA-BDS reduced the friction and wear for all the tested concentrations. H_2_O + NA-BDS exhibited the best lubrication performance. At the optimum concentration of 0.2 wt %, the friction coefficients and wear rates of H_2_O + NA-BDS were reduced by 52.5% and 66.9%, respectively, compared with that lubricated using H_2_O. This indicates that, at different amounts, BDS and NA-BDS can improve the friction and wear behaviors of H_2_O, and that NA-BDS is more effective than BDS.

**Figure 7:**
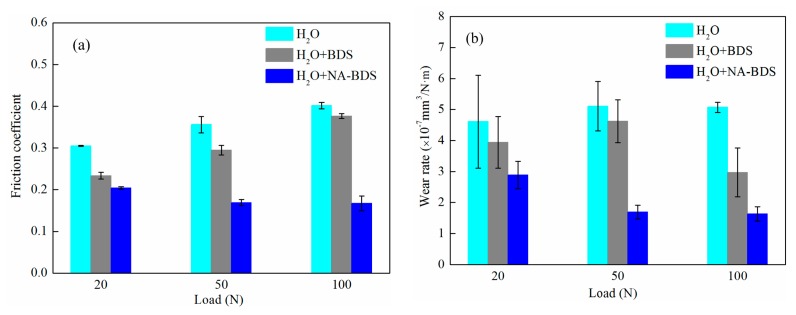
Evolutions of the (**a**) average friction coefficient and (**b**) wear rates for lubrication using BDS and NA-BDS in H_2_O at different loads (soot content = 0.2 wt %, speed = 50 mm/s, duration = 30 min).

The evolutions of the average friction coefficient and the wear rates for lubrication with the two types of soot in H_2_O at different loads are shown in Figure 7. Under the different loads, the friction coefficients and wear rates decreased in the following order: H_2_O > H_2_O + BDS > H_2_O + NA-BDS. Thus, the anti-friction and anti-wear properties of H_2_O + NA-BDS were better than those of H_2_O + BDS and H_2_O. Furthermore, BDS and NA-BDS enhanced the tribological performance of H_2_O from a low to high load, indicating that carbon nanoparticles (BDS and NA-BDS) entered the rubbing surfaces and performed their function, even under a high load.

**Figure 8:**
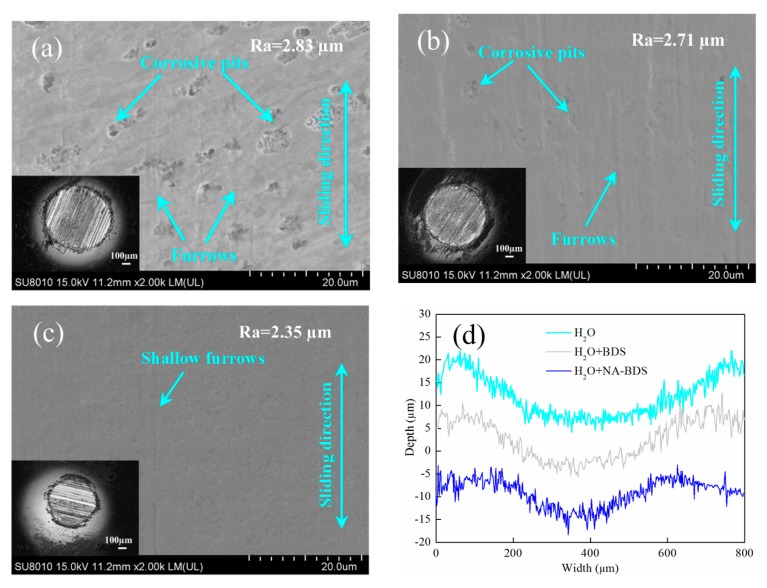
Field-emission scanning electron microscopy (FESEM) images and optical micrographs of wear scars on upper balls lubricated using (**a**) H_2_O, (**b**) H_2_O + BDS, and (**c**) H_2_O + NA-BDS. (**d**) Corresponding worn surface profiles (soot content = 0.2 wt %, load = 100 N, speed = 50 mm/s, duration = 30 min).

Figure 8 shows FESEM images, optical micrographs, and corresponding worn surface profiles of the wear scars on the upper balls lubricated using H_2_O, BDS + H_2_O, and NA-BDS + H_2_O. Comparing Figure 8a–c reveals that the surface wear scars in the case of H_2_O had many corrosive pits and furrows [34]. When 0.2 wt % BDS was added to H_2_O, although the surface wear scars still exhibited many furrows, the number of corrosive pits was reduced. The worn surfaces of the upper balls lubricated using NA-BDS + H_2_O exhibited thin and shallow furrows and no corrosive pits. Compared with H_2_O and H_2_O + BDS, when H_2_O + NA-BDS was used as the lubricant, the surface roughness *Ra* of the worn surface of the ball was lower. Moreover, Figure 8d, which combines the optical micrographs in Figure 8a–c, indicates that the width and depth of the wear scar decreased in the following order: H_2_O > H_2_O + BDS > H_2_O + NA-BDS. This indicates that H_2_O + NA-BDS had a better wear-resistance effect than H_2_O + BDS, in agreement with the results in Figure 7.

### 3.3. Tribological Performance of Soot Samples in LP

**Figure 9:**
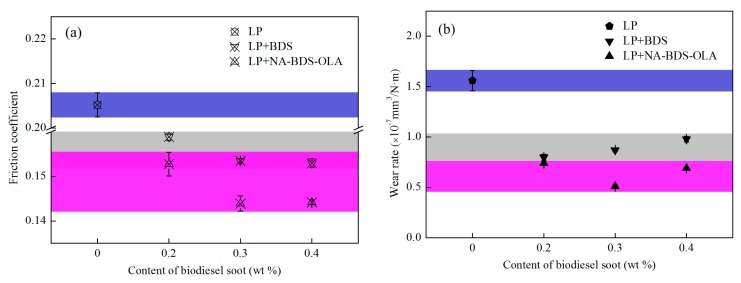
Evolutions of the (**a**) average friction coefficient and (**b**) wear rates for lubrication with different amounts of BDS and NA-BDS-OLA in LP (load = 100 N, speed = 50 mm/s, duration = 30 min).

The evolutions of the average friction coefficient and the wear rates for lubrication using different amounts of the two types of soot added to LP are presented in Figure 9. The friction coefficients and wear rates for LP + NA-BDS-OLA and LP + BDS were lower than those for LP. LP + NA-BDS-OLA exhibited the best tribological properties. At the optimum concentration of 0.3 wt %, the friction coefficients and wear rates of LP + NA-BDS-OLA were 29.8% and 67.3% lower than those of LP, respectively. This indicates that at different amounts, BDS and NA-BDS-OLA can be used as lubrication additives for LP, and that LP + NA-BDS-OLA has better anti-friction and anti-wear properties than LP + BDS.

**Figure 10:**
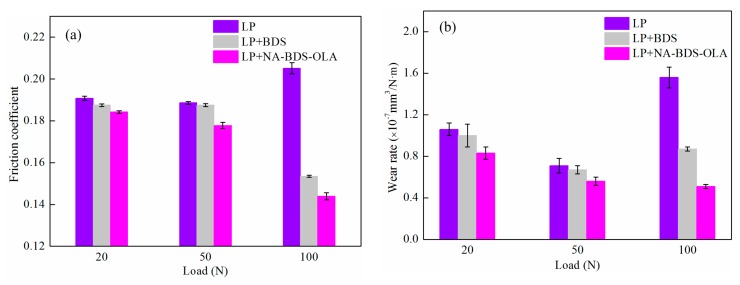
Evolutions of the (**a**) average friction coefficient and (**b**) wear rates for lubrication using BDS and NA-BDS-OLA in LP at different loads (soot content = 0.3 wt %, speed = 50 mm/s, duration = 30 min).

Figure 10 shows the evolution of the average friction coefficient and wear rates for lubrication using the two types of soot in LP at different loads. The results indicate that the friction coefficients and wear rates for LP were the highest, followed by LP + BDS, and then LP + NA-BDS-OLA. Thus, LP + NA-BDS-OLA exhibited the best tribological performance. Interestingly, at a low load (20 N, 50 N), the lubricating property of LP was slightly improved with the addition of BDS and NA-BDS-OLA. However, at a high load (100 N), the tribological properties of LP were significantly improved by adding BDS and NA-BDS-OLA. These results imply that an LP lubricant film forms at a low load and is easily damaged at a high load [35], possibly owing to the increased friction and wear of the LP under a high load. BDS and NA-BDS-OLA can play a positive role in protecting the friction interfaces [36]; hence, they have favorable anti-friction and anti-wear effects, improving the LP tribological properties under a high load.

**Figure 11:**
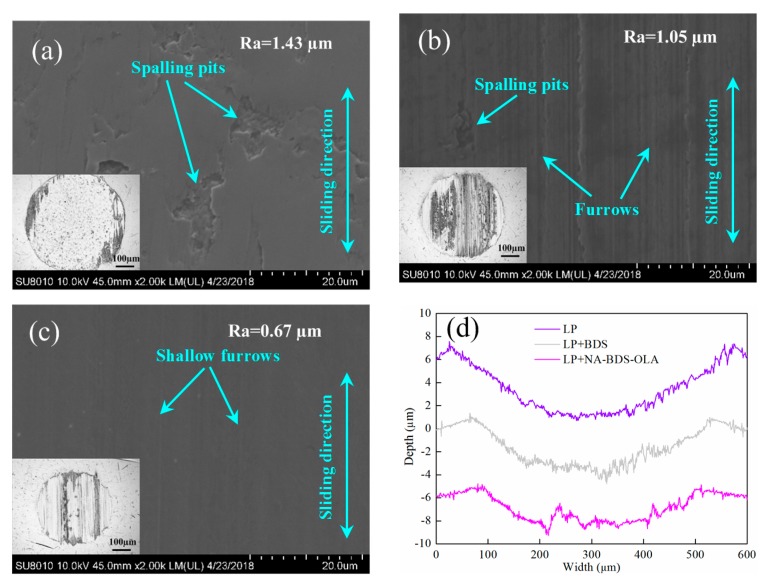
FESEM images and optical micrographs of wear scars on upper balls lubricated with (**a**) LP, (**b**) LP + BDS, and (**c**) LP + NA-BDS-OLA. (**d**) Corresponding worn surface profiles (soot content = 0.3 wt %, load = 100 N, speed = 50 mm/s, duration = 30 min).

Figure 11 shows FESEM images, optical micrographs, and the corresponding worn surface profiles of wear scars on upper balls lubricated using LP, LP + BDS, and LP + NA-BDS-OLA. Compared with the FESEM images in Figure 11a–c, the worn surfaces lubricated with LP exhibited many clear spalling pits. Numerous serious furrows and small pits were observed on the worn surface in the case of LP + BDS. For LP + NA-BDS-OLA, the worn surface exhibited no spalling pits and shallower furrows. The surface roughness *Ra* of the worn surface decreased in the following order: LP > LP + BDS > LP + NA-BDS-OLA. According to the optical micrographs in Figure 11a–c and the corresponding worn surface profiles of the wear scars in Figure 11d, the width and depth of the wear scars decreased in the following order: LP > LP + BDS > LP + NA-BDS-OLA. This indicates that the main type of wear in the case of lubrication with LP was fatigue wear [37], whereas for LP + BDS, the fatigue wear was reduced. However, abrasive wear was observed on the worn surface. Because BDS can fill the friction pairs and act as a balling bearing, the aggregation of BDS can cause abrasive wear [38,39]. Apart from the spacing and ball bearing effect, the carbon onion-like nanoparticles (NA-BDS-OLA) possibly alleviated the friction shearing through their exfoliating behavior [40], which is in accordance with the results shown in Figure 10.

### 3.4. Lubrication Mechanisms

**Figure 12:**
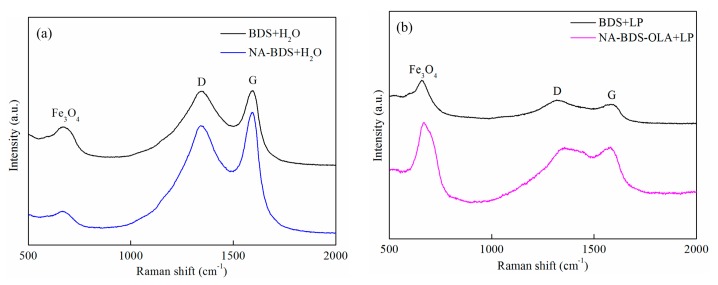
Raman spectra of the worn surfaces of upper balls lubricated using (**a**) BDS + H_2_O and NA-BDS + H_2_O, and (**b**) BDS + LP and NA-BDS-OLA + LP.

Raman spectroscopy can be employed to obtain detailed information regarding the level of order in the structure by investigating the D and G peaks. The correlation between the structural changes of soot and the Raman spectroscopic parameters has been discussed in the literature [41,42]. Therefore, the order of soot before and after the friction test must be considered in Raman spectroscopy analyses. To investigate the differences in the friction reduction and wear resistance between BDS and NA-BDS, and between BDS and NA-BDS-OLA, we performed a Raman spectroscopy analysis of the wear surfaces. The Raman spectra of the worn surfaces of the upper balls are shown in Figure 12.

The characteristic spectra of the D and G peaks were observed for the worn surfaces, indicating that the carbon nanoparticles deposited on the sliding surfaces formed a protective film. According to the Raman results in Figure 2 and Figure 12, compared with the I_D_/I_G_ ratio for BDS (2.956), that of the worn surface lubricated using BDS in H_2_O (2.986) was not significantly increased, whereas that of the worn surface lubricated using NA-BDS in H_2_O (2.898) was significantly higher than that for NA-BDS (2.546). The peak at 660 cm^−1^ is attributed to Fe_3_O_4_, which formed a protective tribofilm through tribochemical reactions [43,44]. Moreover, the I_D_/I_G_ ratio for BDS (2.956) was similar to that of the worn surface lubricated using BDS in LP (2.927). However, the I_D_/I_G_ ratio for the worn surface lubricated using NA-BDS-OLA in LP was 2.700, which was higher than that for NA-BDS-OLA (2.559). An Fe_3_O_4_ (660 cm^−1^) tribofilm was created during the sliding [45]. These results imply that the formed Fe_3_O_4_ is capable of acting as a lubricious oxide. In contrast to BDS, NA-BDS and NA-BDS-OLA were prone to being exfoliated, generating isolated graphitic layers under a high load, which reduced the friction and wear [40].

According to the foregoing analysis, BDS can provide spacing and act as a ball bearing between the rubbed surfaces. However, NA-BDS and NA-BDS-OLA are structurally similar to onion-like carbon and have significantly higher degrees of graphitization order than BDS. The lubrication mechanisms of NA-BDS as a lubricant additive to H_2_O and NA-BDS-OLA as a lubricant additive to LP can be summarized as follows: First, both types of soot can penetrate into the friction pairs and be deposited on the sliding surface, thereby playing a positive role in reducing the direct contact between the micro-convex bodies during the friction experiment [38]. Second, both types of soot can act as a ball bearing between the friction pairs [31,46]. Finally, under a high load, the graphitic layers of both types of soot are prone to being exfoliated and adsorbed onto the friction interface, resulting in easy sliding and a good lubrication effect between the surfaces [36,47].

## 4. Conclusions

NA-BDS treated with nitric acid and NA-BDS-OLA modified with OLA were used as lubricant additives to H_2_O and LP, respectively. Raman spectroscopy and FETEM indicated that both types of soot (NA-BDS and NA-BDS-OLA) exhibited a better order than BDS, having onion-like structures with a size of 35–40 nm. FTIR and particle-size distribution analysis revealed that NA-BDS and NA-BDS-OLA introduced strong hydrophilic and lipophilic groups, respectively, which reduced the average particle size.

To evaluate the tribological performance of the carbon onion-like nanoparticles derived from BDS, the tribological properties of NA-BDS in H_2_O and NA-BDS-OLA in LP were investigated. We found that the carbon onion-like structure of the two types of soot reduced the friction coefficients and wear rates. The tribological mechanisms can be divided into three mechanisms: (a) a spacing effect, where both types of soot act as spacers between the contact surfaces; (b) a ball bearing effect, in which both types of soot create a rolling between the friction pairs; and (c) an exfoliation effect, where a high load contributes to the formation of exfoliated graphitic layers from the two types of soot during sliding. These graphitic layers can be adsorbed onto the friction surfaces, resulting in easy sliding.

## Figures and Tables

**Table 1:** Physical properties of the biodiesel.

Property	Value	Methods
Density at 20 °C, kg·m^−3^	875	ASTM D4052
Flash point (closed up), °C	168	ASTM D93
Acid value, mgKOH·g^−1^	0.045	ASTM D664
Kinematic viscosity at 40 °C, mm^2^·s^−1^	4.5	ASTM D445
Water content, %	Trace	ASTM D1744
